# Occupational Stress in Chinese Higher Education Institutions: A Case Study of Doctoral Supervisors

**DOI:** 10.3390/ijerph19159503

**Published:** 2022-08-02

**Authors:** Xueyu Wang

**Affiliations:** College of Foreign Languages, Huaqiao University, No. 269 Chenghua North Rd., Quanzhou 362021, China; susan9686@163.com

**Keywords:** higher education, supervisors, distress, factors, grounded theory

## Abstract

This qualitative study is intended to explore the factors that contribute to the occupational stress suffered by Chinese doctoral supervisors and the kind of measures needed to effectively address the issue. Through purposive and snowballing sampling, 30 Chinese doctoral supervisors in different disciplines of natural science and social science were selected. A semi-structured interview protocol was used, and the data were analyzed based on grounded theory methodology. Chinese doctoral supervisors experienced varied stressors of nuanced nature, which could be categorized into two core categories, i.e., performance-appraisal-related factors and Ph.D. student-related factors, which were further divided into 18 subcategories and 10 higher-level categories. Chinese doctoral supervisors are under various sources of stress, corroborating with and reinforcing previous research findings in respect to occupational stress worldwide. Through the analysis of the stress triggers, suggestions are presented in regard to what mental health professionals and educational policy makers can do to address the issue of concern for doctoral supervisors.

## 1. Introduction

Research worldwide reveals that occupational stress and workplace health has been a widespread epidemic for all genders and ages, and has become an issue of great concern over the last decade, both internationally and nationally [1]. This study adopts the definition of “stress” by the Canadian Mental Health Association, i.e., it is a reaction to a situation—it is not about the actual situation. In other words, people usually feel stressed when they think that the demands of the situation are greater than their resources to deal with that situation (https://www.ccohs.ca/oshanswers/psychosocial/stress.html, accessed on 18 December 2018). Given the value of work in this society, the amount of time spent at work and the current changes that are affecting the nature of work, it is not surprising that work stress appears to be increasing [2].

This is especially the case in educational institutions. Apart from various reports about student stress [3,4], a brief review of the literature shows the prevalence of stress, anxiety and depression among teaching staff. For instance, the 1998 and 2004 surveys in British universities revealed that, compared with other professions in the United Kingdom, the academic staff were experiencing higher levels of psychological distress, due to the increasing responsibilities and the high demands of their jobs, and there had been little betterment in the work standards of health and safety within six years [5]. Meanwhile, a study of the 1086 employees of a Quebec university in Canada found that the level of psychological distress reported from individuals was twice as high (40%) as that in a Quebec-wide sample (20%) [6], echoing the findings of the prevalence of occupational stress, anxiety and depression among Egyptian teachers in another study, due to such independent predictors for depression as inadequate salary, higher qualifications, workload, etc. [7].

In China, research also reveals that stress, which causes a chain of psychological problems, is not only epidemic among primary and secondary school teachers, closely attributing to gender, school types, marital status, physical health, economic situation, etc. [8], or professional pressure [9], but also widespread among university teachers, due to sense of value, sense of meaning, sense of identity and sense of helplessness [10] or due to scientific research performance pressure and their occupational stress and anxiety [11]. These findings, along with other reports, reveal a striking fact that in China, there is a tendency for academic scholars to die young, i.e., quite a few prominent scholars died unexpectedly in their prime, which to an extent indicates stress is prevalent among academia (https://www.zhihu.com/question/334804133, accessed on 26 July 2020).

Although a growing line of research worldwide has examined mental health problems in academic contexts and this undertaking has clearly yielded a range of important insights, and internationally, there is an increased emphasis on formal training, monitoring and accountability of doctoral supervisors (e.g., [12,13]), little in-depth research has ever been carried out to explore the kind of anxiety or stress that doctoral supervisors experience and why they suffer from stress of this kind or another, except for a limited number of studies which address, respectively, doctoral supervisors’ emotional, professional and intellectual issues through personal, learning and institutional dimensions [14], and supervisors’ anxiety or stress caused by the change in supervisory relationships (i.e., when a supervisor takes on a Ph.D. student previously supervised by another or has to hand over a student to another supervisor’s care) [15].

Regretfully, no research has ever been carried out to explore the stress issue among Chinese doctoral supervisors. However, quite a few news reports about higher education in recent years have highlighted the importance of the stress issue among doctoral supervisors. For example, on 7 July 2019, a professor and doctoral supervisor at Tsinghua University, died at the age of 56 (http://www.bjnews.com.cn/edu/2019/07/09/600979.html, accessed on 9 July 2019); in October 2020, a female professor of history at Fudan University died at the age of 42 (https://new.qq.com/rain/a/20201012A0DZ1300, accessed on 12 October 2020). They are but a few of those scholars who died in their forties or fifties, so rumor has it that fifty is a doomed age for academics. Further, in 2016, a professor of literature and doctoral supervisor at Xiamen University, after being disqualified from being a supervisor due to his failure to comply with the university policy governing the recruitment process for Ph.D. students (http://news.sohu.com/20160224/n438383997.shtml, accessed on 24 February 2016), published an open letter proclaiming that he had decided to leave academia.

These cases could be just a tip of the iceberg. More cases and reports popping up time and again in social media highlight the psychological stress experienced by Chinese doctoral supervisors, attracting more concerns and attentions from the public. Doctoral supervisors are usually those limited individuals who enjoy a high-level reputation for their academic achievements in certain areas. For the public, the title of “supervisor” is not only a symbol of academic awards, but also a symbol of prominent social status. But few people could imagine that under this glory, these individuals’ mental health is eroded bit by bit due to the overwhelming stress. The striking silence about the prevalence and determinants of mental health problems among Chinese doctoral supervisors necessitates a further study to address this gap in the doctoral education literature.

## 2. Materials and Methods

Research methods applied in previous studies range from quantitative to mixed approaches, and instruments are adopted for statistical analyses and calculation of stress levels (e.g., [6,7]). However, the purpose of this study is to explore the factors that contribute to the occupational stress suffered by Chinese doctoral supervisors. So qualitative research methods are appropriate for in-depth investigation on this issue, and among them, grounded theory is helpful to understand how and why Chinese doctoral supervisors are experiencing stress.

### 2.1. Research Design

Grounded theory (GT), a well-established methodological and qualitative approach for context-specific inductive theory building or an inductive enquiry that explains social processes in complex real-world contexts [16], is favored in this study, as it provides a major contribution to the generation of emergent theory when there is little known about a particular phenomenon. GT is applied based on the Strauss and Corbin approach [17]. As such, it is necessary to conduct grounded theory, in order to understand the stress-related experiences of doctoral supervisors and establish some countermeasures.

A pilot study was conducted to serve such purposes as adding value and credibility to the semi-structured interviews to be used in the major study, avoiding inefficient theoretical sampling or even erroneous purposeful sampling and providing remedial loops, or effecting changes of focus, guidance to improve data collection instruments, and/or informing theoretical sampling [18], or avoiding certain ethical problems [19], among others. Specifically, initial interview questions were first designed pertaining to the stressors that supervisors may endorse (Table 1), then open-ended questions were emailed for expert reviews in the interview protocol. The broad and general initial interview questions were revised, which was based on the suggestions from the experts, in order to prepare for defining and clarifying the research theme in the pilot testing. After that, participants were recruited from the selected universities and the pilot interview was conducted. Purposeful sampling was adopted in the selection of the informants, so that the participants in the pilot interview shared as similar criteria as possible to the group of participants in the major study [20,21]. Two doctoral supervisors, in their 40s and 50s, were interviewed in their offices in October 2021. The two interviews, including the social conversation to build rapport as suggested by Jacob and Ferguson [22], ranged in time between approximately 35 min to 45 min and were recorded using a smart phone. Both of them were informed of the research goal and were willing to participate in it.

Upon completion of the initial interview, the data were transcribed and analyzed to deduce themes and concepts. The analysis of research participants’ response to the questions laid a basis for the questions to be used in the follow-up interviews. As themes emerged, the interview questions for the second round of interviews were narrowed down and focused on the existing themes. In this way, new themes emerged while the existing themes were confirmed. So, the follow-up interviews were conducted to confirm or clarify emergent themes, and meanwhile, to explore any new information. The pilot study helped to test the appropriateness of the interview questions, and adjust the research plan, identify the possible problems, obtain experience and build rapport in the follow-up in-depth, semi-structured interviews in the major study.

### 2.2. Participants and Ethical Considerations

Throughout the process of participant recruitment, purposeful and snowball sampling was used, i.e., early recruits led to the later recruits. The participants consisted of 15 men and 15 women who ranged in age from 33 to 62 at the time of the interview, came from northwestern, southwestern, central, northeastern and southeastern areas of China, and majored in varied disciplines of natural science and social science. Except one who had a Master’s degree, the rest of the participants had doctoral degrees. Participant selection, data collection and data analysis continued until theoretical saturation reached. Data collection ceased after 25 interviews, as it was clear that no new themes emerged; but 5 additional participants were interviewed to reduce the chance of missed themes and ensure that data saturation was achieved.

Prior to the commencement of the interview, an initial explanatory statement was sent via WeChat to identified supervisors who were informed about the purpose of the study, the length of the taped interview (approximately 35 to 45 min in duration), the voluntary and confidential nature of the interview (i.e., any personally identifiable information, such as their names and affiliations, would be removed or changed before research findings were shared with other researchers or results were made public), the way of answering the study instruments, their right of withdrawal or termination (i.e., they were free to withdraw from the research at any point after initially consenting to participate) and written consent for participation was sought from all participants. Further, official approval was sought from pertinent authority, so as to comply with pertinent codes of ethics (e.g., [23,24,25]).

### 2.3. Interview and Data Collection

At the outset of the interviews, participants were reassured of anonymity and confidentiality of the interview, helping to build trust [26], and semi-structured in-depth interviews were used for its varied functions, such as encouraging depth and vitality [27,28] or soliciting new concepts to emerge [29]. Due to the novel coronavirus disease (COVID-19), interviews were conducted via WeChat during the past few months. After a brief introduction to his/her personal information, each participant answered the open-ended questions, which facilitated a free flow of ideas from the respondent and generated information-rich data. Meanwhile, a clinical method for interviewing was adopted [30]. Thus, the participants were requested to elaborate on a statement if it was vague or ambiguous, so as to ensure the clarity of certain concepts.

Each interview was conducted in Chinese and audio recorded by the in-built audio recording software of WeChat. Each participant was assigned a code number to avoid revealing his or her identity. A professional transcriptionist was hired to transcribe the audio files into Mandarin Chinese, and each interview transcript was under a first-pass verification, i.e., verified by each participant for accuracy and validity, then the second-pass review was conducted by the interviewer using the original audio files and anonymized, formatted transcripts, to complete a final check of content accuracy and then adjust the level of transcription for analysis. After the transcripts were double checked, they were translated into English.

In qualitative research, interview translations always aim for semantic equivalence at a minimum and aspire to conceptual equivalence [31]. To validate the translation quality and demonstrate the functionally equivalent translation, the back-translation method was adopted, as suggested by Brislin [32].

After verifying the trustworthiness or credibility and increasing the accuracy of the study through triangulation, i.e., participant checking, self-reflection and peer review [33], the transcripts and the translations thereof were stored and analyzed side by side. A format came into being that helped the research team to discuss the transcripts and interact with the original and translated versions [34]. Data coding was conducted by language-congruent researchers in the source language, adjacent to the same data in the English translation, as recommended by Olson [35].

### 2.4. Data Analysis Method

Based on the questions in the interview guide, verbatim transcripts were read and analyzed repeatedly to identify themes related to stress, then filtered and selected through the constant comparative method. The whole process involved three phases of coding (i.e., open, axial and selective coding) [17]. First, the stress factors experienced by supervisors were identified by dismantling and reviewing the data collected through open coding to form codes, then the codes were categorized. Second, all the higher order codes were merged, and data were grouped and linked to each other through axial coding. The relationships between categories were organized into a paradigm model that includes, causal conditions, contextual conditions, interventional conditions, action-interaction and consequences. Third, the core categories were identified through selective coding, thus leading to a substantial theory that could explain the stressors experienced by Chinese doctoral supervisors (Table 2).

## 3. Results

**Figure 1:**
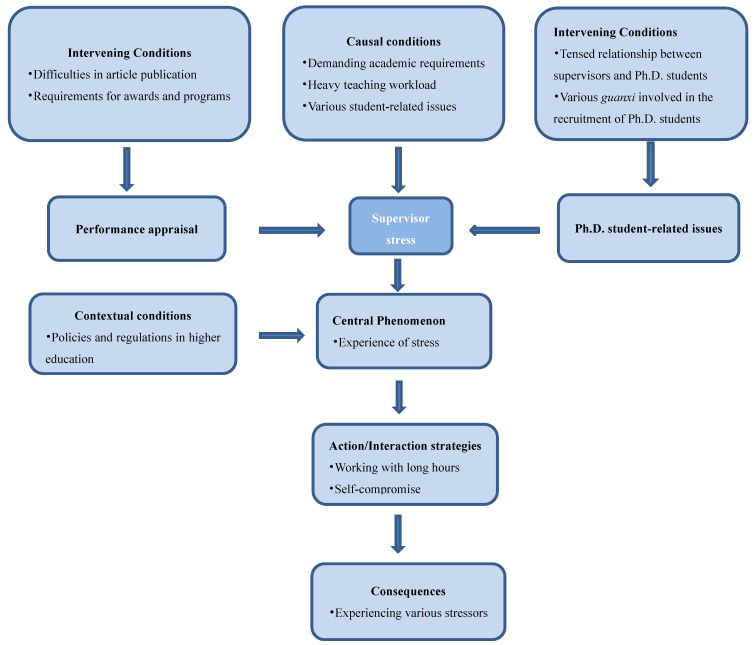
Paradigm model of occupational stress suffered by Chinese doctoral supervisors.

The participants revealed a variety of factors contributing to this widespread stress among Chinese doctoral supervisors. Nevertheless, the GT emerging from the data represented the nuanced nature of participants’ experiences with stress. Categorization of the interview data through open coding yielded 18 subcategories and 10 higher-level categories. The relations of categories were linked through axis coding according to the paradigm model (Figure 1). Data analysis was found to comprise two core categories, i.e., performance appraisal and Ph.D. student-related issues.

### 3.1. Causal Conditions

The participants were found to be engaged in “demanding academic requirements”. First, it was extremely tough, if not totally impossible, to publish three or more articles in such designated journals indexed by SCI, SSCI or CSSCI (the abbreviation of Chinese Social Sciences Citation Index “中文社会科学引文索引”, herein after referred as “*hexin* journals”, meaning “key journals”) during the contract term of three or four years. Second, the requirement to host funded research program at ministerial level or above increased the stress for the participants, although for some participants, it was an alternative to article publication requirement.

“…You know, almost all universities in China developed a Guide for Journal Category. Category D1 journals include the rest of journals covered in CSSCI except for Category A, B and C journals. My university adopted the similar journal categorization system that CSSCI journals are categorized into A1, A2, A3…; B1, B2, B3… To get three articles published in these Category C journals, or two articles in Category C journals and two articles in Category D1 journals in three years, is absolutely a fable, at least to me.”

(Participant 12)

“…Winning any award or hosting a *NSSFC* (National Social Science Fund of China) or *NSFC* (National Natural Science Foundation of China) is tantamount to finding a needle in a haystack.”

(Participant 3)

The interview data also revealed that the supervisors had to undertake “heavy teaching workload”. Different from the credit hour adopted in western countries, the workload (工作量), as devised by the Human Resources of varied Chinese universities, comprises the number of equivalent 40 (45, or 50)-min lecture periods and credits for supervising laboratories or supervising graduates per semester. Some universities require that supervisors independently teach at least one undergraduate level course and supervise a certain number of postgraduates per year. The annual workloads of the participants ranged from 300 to 340 credit hours in two semesters, which is equivalent to teaching five to six courses per semester, namely, 10 or 12 credit hours per week for 16–18 weeks. A minimum of their annual workloads, e.g., 120 credit hours of undergraduate-level teaching is required, depending on their respective salary range and position grade they have contracted with their universities.

“As a full-time professional, the workload is too much for me. It is tough to strike a balance between academic research and teaching.”

(Participant 4)

Further, the supervisors had the end-of-course teaching evaluations by students via a Management Information System at the end of each semester. This system is run by the University, also called teacher performance appraisal system (TPA). It is a 100-point scale questionnaire. Students are instructed to log in to fill out the TPA form online. The evaluation ratings for teachers should reach a minimum score of 90 for teachers to satisfy the requirements set for the position of their respective salary grade.

“…student appraisal is always affected by some unexpected or irritating factors…e.g., low scores will result in retaliation from students…”

(Participant 2)

As for “various student-related issues”, the participants reported that student graduation with article publication and thesis completion was the key source of their stress. Moreover, the supervisor–student relationship was an unexceptional trigger for stress. With regards to new Ph.D. student recruitment, not only the unspoken rule or *guanxi*, but the number of recruitments required would be stressful.

“If a student can’t publish an article in a *hexin* journal as required by the University, he/she can’t graduate on time. Further, he or she must compose a thesis (either a monograph or compilation), undergo external review of thesis, and pass the public defense of dissertation for Ph.D.”

(Participant 13)

“…Some of my students seldom clean up laboratory glassware or other instruments after they finish experiments, and reluctantly comply with experiment regulations or protocols, although I remind them time and again about the importance of following the rules.”

(Participant 11)

“…Sometimes, it is really hard to strike a balance between those varied *guanxi*, especially when I have some adoring students who decide to pursue a doctoral degree. As such, I often maneuver into a peculiar dilemma as to which student to recruit.”

(Participant 16)

### 3.2. Central Phenomenon

All the participants shared “negative emotions” throughout the interview process. The pervading gloominess, sullenness or stress was obvious or pervasive when they mentioned that their articles had been repeatedly rejected by varied journals. Some participants were wondering whether they were psychologically healthy, saying that they could not imagine gloating over other people’s failure. In a sense, article publication involves losing or gaining face.

“…one of my daily routines is to ask whether my colleagues have got article rejected, accepted or published. Negative response would console or excite me, while positive response would make me jealous and more distressful.”

(Participant 15)

“…Supervisors are supposed to have publications in high impact factor journals, and to inspire the students as a role model and command respect.”

(Participant 6)

All participants also remarked that they were always in a paradox, i.e., if their grant proposals were rejected, they would feel heartbroken, but being granted a funding program was nothing more than a short-lived pleasure of success, or just the beginning of another disaster.

“…I would always be on tenterhooks before program approval is published. When your application is rejected, you would think the sky is about to fall down.”

(Participant 5)

In all, 8 out of 30 participants remarked that their workload was rather heavy and exhausting. As for teaching evaluations, although 21 participants seemed not bothered by it at all, 9 participants deemed it as a major stressor.

“The assigned workload makes me worn out, and I easily get irritated even over trifles in daily life.”

(Participant 4)

“…you know, a low rating for your teaching not only means loss of money, but more importantly, loss of face.”

(Participant 18)

During the interviews, all the participants shared the view that thesis and article publication issues were key sources of stress, for failure to meet these requirements not only jeopardizes students’ future (namely, rejected doctorates), but also exerts significant negative impacts on their supervisors, e.g., illegitimacy to recruit new Ph.D. students and loss of face, among others.

A total of 13 out of 30 participants mentioned their stressful experience with students. Half of the participants proclaimed to have been stressed by the unspoken rule or *guanxi* issue in the process of recruiting new Ph.D. students.

### 3.3. Contextual Conditions

The participants reported that their stress was mostly attributed to “policies and regulations in higher education”, one of which was performance appraisal. As a rating mechanism, performance appraisal is employed by almost all universities and institutions of higher education. In this way, job performance of an employee is evaluated in terms of quality, quantity, cost and time, and human resources development provides continued push for personnel accountability and competency and effective schooling, and determines who needs what training, and who will be promoted, demoted, retained or fired [36]. As dictated by the participants, performance appraisal, as a major stressor, consisted of such rating factors as article publication, research program and teaching.

“It is quite unreasonable to publish 3 or more articles in key journals, or host a research program at ministerial level or above during 4 years. Moreover, I have to undertake 340 workloads in two semesters. Otherwise I will be degraded or removed from the current post.”

(Participant 17)

According to the supervisor’s guide promulgated by most universities, supervisors should have adequate funding to allow the research to progress (this is especially the case for supervisors of doctoral students in science and engineering), and having adequate funding (e.g., RMB 200,000 or RMB 400,000) was the prerequisite for new Ph.D. student recruitment. Consequently, supervisors were desperate for grant programs, availing every effort to draw funds from all sorts of corners to support their students’ research.

“Hosting a NSSFC or NSFC or winning those awards not only helps satisfy the requirements for my salary range and position grade, but also provides additional funding to take on new students.”

(Participant 21)

As for student-related issues, student graduation comes first. In China, the total duration for completion of degree requirements and submission of thesis for external evaluation is three regular years, or a maximum of six years, as of the date of admission as a Ph.D. student, during which time each candidate shall complete required credits in a subject, and publish, out of his or her research work, at least one research publication (letter of acceptance included) in a university approved *hexin* journal of his or her research field. Further, he or she should accomplish a thesis, which should make a distinct contribution to knowledge and show ability to conduct original investigations and to test ideas whether of his own or of others, undergo external review of the thesis and pass an oral defense.

“If my students can’t meet the requirements, they have to extend their timeline of graduation. It will affect the recruitment of new doctoral students. What’s worse, I will be disqualified from being a supervisor.”

(Participant 23)

In addition, universities are prone to adopt a policy in favor of students whenever there is supervisor–student confrontation or conflict, as evidenced by numerous cases where Ph.D. students suffered psychological stress, or committed suicide due to pressure from study, but their supervisors were criticized for pressing them too much. As remarked by most participants, supervisors were supposed to take responsibility for everything ranging from Ph.D. students’ daily lives to their graduation, otherwise they would be criticized for omission. As a result, supervisors dare not blame or criticize students, although students are lazy and refuse to follow the instructions or abide by certain academic ethics.

“…to criticize students is to ask for trouble…when a student broke down an expensive instrument due to non-compliance with laboratory regulations, I was irritated. But the most stressful thing was that I could not claim compensation, lest it should cause any stress to him and I get punished in turn. Today is yesterday’s pupil.”

(Participant 11)

In terms of the recruitment of new doctoral students, *guanxi* plays a very important role in it.

“I would like to recruit older candidates from the legal profession for their rich experience in law, but could not always be away from worldly wisdom.”

(Participant 19)

Further, Participants 10, 21 and 27 mentioned the number of Ph.D. students required to recruit was a stressor. The Ministry of Education sets a limit or quota for Ph.D. student recruitment in different subjects or disciplines for each year. As such, in some subjects, a supervisor may recruit one or two doctoral students per year, while in other subjects, two or three supervisors have to share one doctoral student each year.

### 3.4. Intervening Conditions

The participants were found to be faced with various stressors, including “personal performance appraisal” and “doctoral student-related issues”. All the participants had difficulties in publishing articles in key journals within the term of performance appraisal, usually 3 or 4 years.

“The publication threshold for me is 3 articles in Category C journals. Of the 500 journals covered in the CSSCI database, only 190 journals fall into Category C and above. But of the 190 journals, only 10 journals fall within my research field or discipline…You may say that there are some SSCI indexed journals…you know, it’s even harder, for you have to compete with academics worldwide. What’s worse, only the top 25% of SSCI indexed journals in one particular discipline are deemed as Category C, in accordance with the Journal Category Guideline devised by my university.”(Participant 7)

Apart from publishing the designated number of articles, the participants reported that it was tough to meet the requirements to win awards or host research programs at ministerial level or above, from the beginning of application to the deadline.

“…it is one thing to be great at your job, but quite another to win any of these awards… you must have good connections, otherwise your application cannot even go beyond the university. You know, candidates for these awards are firstly recommended by the schools to the university, then recommended to the provincial or ministerial department for deliberation.”

(Participant 25)

“…you have to compose a final performance report which adheres to the Grant Program Guideline, then submit it to the funding authority before the program is due, failing which, you cannot apply for any research program for a couple of years…”

(Participant 8)

The participants also shared the idea that a tensed supervisor–student relationship was a stressor, including:

(a) Students’ complaints about supervisor bias or omission,
(b) Students’ low caliber or non-commitment to research (including indolence, lack of necessary expertise in carrying on experiments, plagiarism, misattribution, dispute over co-authorship, noncompliance with academic ethics, etc.),
(c) Students’ request to change supervisors due to dislike for each other, and/or
(d) other confrontations or noncooperation in relation to their study or research programs.

“Some students complain about my omission in their article publication, claiming that coloration, availability of funds and facilitation in article publication are the key features that must be contained in an active and productive Ph.D. supervisor…it seems as if I have been grossly at fault for their manuscript rejection…”

(Participant 9)

One more stressor is related to various *guanxi* involved in the recruitment of new doctoral students.

“…if an applicant finds that someone (e.g., a colleague, family member, or friend) is well connected to me, the applicant will normally request the contact(s) to refer or introduce him to me, or the contact(s) would ask me to do the applicant a special favor. Where there are two or more applicants, more contacts would come to me.”

(Participant 14)

### 3.5. Action/Interaction Strategies

The participants reported that in order to meet with the requirements of performance appraisal, they had to “work with long hours”. They devoted nearly all their time and energy to academic research and teaching, wondering all day how to deliver excellent on-the-job performance and fulfill their on-the-job responsibilities, so much so that they spent less time on vacation or other leisure activities. Apart from article publication, they had to strive to succeed in:

(a) Hosting a NSSFC or NSFC
(b) Winning an *Award for Scholarly Achievement* (科研成果奖) (at least the Third prize) at national, ministerial or provincial level,
(c) Winning an *Award for Teaching Achievement* (教学成果奖) (at least the Third prize) at Ministerial or Provincial level, or
(d) Becoming a *Talent Excellence Program trainee* (优秀人才项目) at ministerial or provincial level.

“…(being granted a funding program)what follows is long-lived torture of tedious research work, day and night, in order to produce either books, monographs, peer-reviewed articles, e-books, digital materials, experiment reports, translations with annotations or a critical apparatus, or critical editions resulting from previous research, before undergoing a review by relevant authority comprising of peer scholars.”

(Participant 7)

“I have to undertake 340 workloads in two semesters, and teaching students at undergraduate level shall account for 35% (i.e., 120 workloads) of the total workload. I try to strike a balance between academic research and teaching, always staying up late.”

(Participant 26)

While dealing with student-related issues, most participants claimed that they were liable in opting for “self-compromise”, in order to avoid trouble or reduce the conflicts between supervisors and students.

“…Once, an experiment instrument worth ¥300,000 broke down for the student failed to comply with the obligations specified in the operation protocol. What stresses me out most is I can’t claim compensation, lest it should cause any stress to him and I get punished in turn.”

(Participant 11)

“…this year, I have to share one doctoral student with a colleague. In the end, I stroke a deal with my colleague, i.e., I gave my half quota to him, so this student is under his supervision this year, and he promised to give me his half quota the following year… this means I can recruit one student every two years.”

(Participant 10)

### 3.6. Consequences

It is noteworthy that despite their divergent perspectives as to age, gender, length of time and experiences in supervising students, etc., the participants “experienced various stressors”, that is, they shared the same or similar psychological stress as an experience associated with their lives as doctoral supervisors; although some were stressed by a single factor, others were stressed by the combination of a set of factors.

“Sometimes I feel frustrated, however, I make full use of my spare time to pursue projects that embody exceptional research, rigorous analysis, and clear writing, carefully and smartly articulate the projects’ value to humanities scholars or general audiences, so that I might edge past the fierce competition and be granted the project.”

(Participant 22)

“…if I get a low rating for my teaching, I will fail the performance appraisal, resulting in deduction of annual salary, or demotion to a position of a lower salary grade as a punishment…”

(Participant 2)

“…having no students to supervise not only impairs the conduction of experiment, but also greatly impacts my performance appraisal.”

(Participant 19)

## 4. Discussion

The GT of supervisor stress, which was developed from the results of this study, indicates that supervisor stress is a multifaceted process inflicted by the influences of varied factors, individually or collectively, and their current stress at the time of the interview represents a result of the ongoing combination of value system, social norms and/or educational policies.

***Apprehension at performance appraisal failure***

Apprehension or angst at performance appraisal failure was viewed by all the participants as the most important trigger for their stress, although the subcategories mattered differently to the participants. Admittedly, it is rather difficult, if not altogether impossible, to publish three or more articles in *hexin* journals, receive funding (from national or provincial governments) for research projects and undertake 300–400 workloads simultaneously. After all, performance appraisal failure entailed not only disqualification for recruiting new Ph.D. students, but also reduced income and loss of dignity or face. This finding corroborates the findings of the most recent DFE (Department for Education) teacher workload survey that heavy workload is one of the biggest threats to teacher recruitment and retention and the level of workload is generating stress in schools [37].

The reason for imposing such harsh performance appraisal standards is closely tied to the tertiary education reforms.

One of the reforms is the scale expansion of Ph.D. students as a countermeasure to release the burden of unemployment rate among college graduates that has been rising in the past few years. The boosting enrollment means that supervisors have to supervise a growing number of Ph.D. students, which consequently contributes to the double dilemma arising from the apprehension at student graduation. Indeed, Chinese supervisors are carrying a supervision load of 5.77 doctorate candidates, much higher than the recommended threshold of 3 Ph.D. and 5 Master’s students in any given academic year internationally. Theoretically, the more students they supervise, the more stressful they would find it. In contrast, in some countries, the average academic has a maximum potential of training 27 Ph.D. students in a 30-year career [38]. As more and more universities around the world graduate ever-increasing numbers of students with doctoral degrees, governments are beginning to ask if it is time to slow the production line [39].

The other reform is the Double First-Class Construction (*shuang yi liu*, 双一流, is short for “The World First-Class University and First-Class Academic Discipline” (Chinese: 世界一流大学和一流学科) Construction). This plan represents a new way of ranking universities in China. Up to date, according to the lists issued by the Chinese Ministry of Education, Double First-Class Universities consist of Class A (including 36 universities), Class B (including 6 universities), and Double Class discipline universities (including 95 universities). It was a tertiary education development initiative designed by the Chinese government in 2015 to develop elite Chinese universities and individual faculty departments into world-class institutions by the end of 2050. Currently, global university rankings attract considerable attention, and such rankings may cause universities to prioritize activities and outcomes that will have a positive effect in their ranking position [40]. The ranking systems affect universities globally [41]. To be shortlisted as a candidate of the Double First-Class Construction not only means being promoted to fame and a better student pool, but also to larger financial subsides and a higher income. As such, when designing a performance appraisal system, almost all universities set high academic and teaching standards, so that the shortlisted universities can maintain their places, while the universities that have not been shortlisted have a chance to enter. According to the Ministry of Education, the list is the result of competitive selection, expert evaluation, government assessment and periodic screening.

All the participants believed the performance appraisal was unreasonable, corroborating other findings that performance appraisal triggers nothing but dread and apprehension and is favored by no one except employers [42,43]. The process may conceal the decision-making and communication function of performance appraisal in organizations, despite the heightened view of appraisal as a measurement tool [44]. The number of publications or a single score on a rating form does not sufficiently measure the contributions of faculty.

***Apprehension resulting from student-related issues***

Of the student-related issues, apprehension at failure to graduation on time was a definite stressor for Ph.D. students. After all, to publish one or two articles in *hexin* journals is tough for supervisors, let alone Ph.D. students, corroborating other findings that doctoral students face high and potentially strenuous demands [45], and article publication is a major stressor for Ph.D. students [3].

Meanwhile, this is also a major stressor for supervisors, because supervisors’ recruitment of new Ph.D. students and performance appraisal would be significantly impaired. The POP (publish or perish) practice is prevalent worldwide, obliging supervisors and Ph.D. students to spend more time scrambling to publish whatever they can get into print [46]. Since this practice entails inevitable ambivalence and resistance among doctoral supervisors and candidates in respect to the place of publication in doctoral work [47] and “mandating publications for graduation places a poor metric on Ph.D. students’ skills and detrimental effects on Ph.D. training…” [48], it is suggested that the POP practice be abolished [49].

In addition, thesis writing is a stressor which not only increases stress for Ph.D. students [50,51], but also causes significant harm or severe emotional distress to supervisors. After all, the qualities of doctoral dissertations and graduation rates are directly related to the Ph.D. student recruitment and performance appraisal of supervisors.

The relationship between supervisors and Ph.D. students is a complex one. The tensed supervisor–student relationship, whether personal or professional, also makes for a major stressor. The interviews revealed that such factors as students’ lack of interests and skill sets, non-cooperation, hostile conflict, mutual dissatisfaction and defiance of academic ethics, may result in tensions and stress to some supervisors, as evidenced by some news report about plagiarism allegation against two Ph.D. students whose supervisors have been temporarily suspended or permanently removed from the Supervisor Register [52]. These are just a few. Stories such as this pop up in the news regularly. For example, Jinan University, Anhui University and Fudan University accused some Ph.D. graduates of plagiarism, and supervisors of those graduates were suspended temporarily or removed from the Supervisor Register (https://zhuanlan.zhihu.com/p/46609954, accessed on 12 October 2018, http://news.sohu.com/20160322/n441458886.shtml, accessed on 22 March 2016 and https://lx.huanqiu.com/article/9CaKrnJZX3P, accessed on 22 January 2017, respectively).

The intricacy of the supervisor–student relationship corroborates with other findings that the supervisor–student relationship may be in part comparable to the one between the physician and his/her patient, and at some point of the journey, develop different expectations of one another [53], or exert negative effects on the supervisors’ reputation, corroborating the findings that academic dishonesty, especially plagiarism, is a global problem that has bedeviled academia and has been viewed as unethical and immoral intellectual thievery that could negatively impact on not only the repute of an academic institution, but the prosperity of a society [54].

Factors that partly contribute to the tensed relationship can be summed up as follows:

(1) Students’ failure in understanding what a doctoral degree means. A Ph.D. is the highest academic degree in China, which requires the command of more specialized academic knowledge in a certain field. Regretfully, some Ph.D. students have a lack of commitment to academic research, or not psychologically and professionally ready for the doctoral study. A survey conducted by *Nature* also reveals the turbulent nature of doctoral research [55]. Ironically however, a Ph.D. is increasingly becoming a springboard to a higher social status or prestige and greater power or resources. Actually, not every Ph.D. student fully understands that the word “philosophy” signifies an individual who has a passion of wisdom and a clear field of academic interest, and has achieved a comprehensive general education in the fundamental issues of the present world. Some of them merely focus on the final crossing of a symbolic boundary represented by the completed thesis and the viva, ignoring the fact that they should perform a “rite of passage” throughout their research education [56]. As such, the above-mentioned factors (such as indolence, noncompliance and noncooperation) are pervasive and consequently contribute to supervisor stress.

A recent blog published by a professor at Fudan University may represent the voice of all the stressed supervisors in China. The professor taunted about the decline in the competencies of postgraduates and Ph.D. students in China and called on his Ph.D. students to “…understand the stress and anxiety of ‘an advanced animal’ (i.e., the professor himself)”(https://baijiahao.baidu.com/s?id=1625877107917853133&wfr=spider&for=pc, accessed on 19 February 2019). Coincidentally, a professor at Shanghai Jiaotong University, who usually revised students’ experiment protocols or articles until two o’clock in the morning, was greatly irritated by a student laziness or lack of academic expertise, and posted in a WeChat Group some sharp words such as “rubbish or garbage, shit” to address the students’ experimental findings. Subsequently, the university delivered to him an official notice of suspension, required him to make a public apology to the student and issued a notice of criticism circulated within the department. These cases, among others, reveal in part the stress, anxiety, helplessness and apprehension confronting supervisors who are under significant pressure to complete some major yet urgent projects. So, if students have no clear conception about the doctorate, the stress will be there waiting for supervisors.

(2) Lack of training on the part of supervisors. Regretfully, in China, there is no mandatory requirement for formal training or monitoring and accountability of doctoral supervisors, except for the obligatory academic requirements, for example, doctoral supervisors should have graduated with doctoral degrees, have published a required number of articles in *hexin* journals, or have been granted research programs at national or provincial level, as evidenced by the Ph.D. Supervision Guide promulgated by most universities in China. Actually, supervisors, especially the newly elected supervisors, undertake no such training prior to the commencement of doctoral supervision. As a result, supervisors are not completely prepared for the role of supervisor and to have insight into the responsibilities that it entails. After all, the supervisor–student relationship varies, while supervision training will make them aware of their roles and responsibilities.

***Proposed suggestions***

Anyway, the findings reveal the prevalence of stress among Chinese supervisors. Ironically, few people are aware that scholars are at greater risk of stress-related illness than police, medics and local authority staff [57]. At a time when student mental health has become the focal concern of most universities, it is surprising that staff well-being has been attracting the least attention [58].

Thus, it is high time that relevant policies were updated. Solutions proposed for the issue include:

(1) Clarifying respective rights and responsibilities

There is no doubt that supervisors should strictly abide by ethics and codes of practice related to their positions, and are highly expected to promote knowledge development and create a research culture during the research process [59]. However, emphasis should be placed on the norms and the innovations of Ph.D. students’ academic research, such as the standards of citing references against plagiarism and the development of original ideas. Thus, it is indispensable for universities and institutions to draw up the specific guidelines for enrollment, supervision and assessment. Supervisors should be authorized to deal with Ph.D. students’ academic performance in a more supportive and flexible way. Further, awards will go to supervisors to enhance their sense of achievement and develop the notion of “showing respect to teachers and promoting education (*zun shi zhong jiao*尊师重教)”.

(2) Establishing a full-fledged performance appraisal system

Nearly all the participants criticized the current policy makers for ignoring the fact that article publication was beyond their control, and suggested that university management adopt more flexible appraisal approaches. Fortunately, the Ministry of Education of China has been committed to the reform of educational assessment, one of the measures being to remove the “five-overemphasis”. On November 8, 2018, the Ministry of Education carried out a campaign against “five-overemphasis” (破“五唯”), that is, the overemphasis on articles, positions, professional titles, certificates and awards (“唯论文,唯帽子,唯职称,唯学历,唯奖项”). Its purpose is to further push the reform of systems within higher education, improve the mechanism of moral education(“立德树人”), reverse the unreasonable evaluation guidance, operate on the appraisal system based on representative works and value the qualities and the influence of the works. In this way, multi-level performance appraisal is on trial at universities and colleges, including teaching, academic research and service, just as Participant 1 suggested that more indicators should be covered in the performance appraisal system, such as the social service of delivering public lectures or hosting hot-line meetings to address psychological issues related to the novel coronavirus disease. The reform values the evaluation of the teaching process and social service based on the annual assessment. The appraisal system not only covers self-assessment from supervisors, but the appraisal from their peers, Ph.D. students and the administrative staff. All these measures can serve to foster increases in personal knowledge of subject matter and effective methods for delivering the knowledge to students, to increase teacher confidence and, in turn, professional competence and to help teachers reach their potential [36]. After all, it is not the sole objective of the performance appraisal to determine who will be promoted, demoted, retained or fired, for this objective will only dilute and weaken the clarity and validity of any appraisal system [60].

(3) Adopting a more reasonable or flexible Ph.D. recruitment and education policy

This includes introducing a mechanism for changing supervisors when a confrontation between a Ph.D. student and his or her supervisor arises, and adopting an *Application and* *Verification* *System* (申请-审核制) under which Ph.D. candidates shall present their works (including articles published in key journals, programs hosted during their graduate study and research proposals) and show their personalities (e.g., independence, commitment to academic research, team work spirit), to convince their supervisor committee before they are admitted as Ph.D. students, and this system allows supervisors to dismiss disqualified Ph.D. students after due process, for example.

As to the enrollment policy reform, it is advisable to refer to the results of performance appraisal as an important decisive factor in the annual enrollment decision. Outstanding supervisors and teams are encouraged to share their experience and take the lead in Ph.D. education. In this way, improved student outcomes (i.e., qualified Ph.D. graduates) and fulfillment of the objectives of Ph.D. education could be ensured.

(4) Designing and implementing a supervisor training program

It is essential to establish a three-level (i.e., national, provincial and university level) training program, and organize pre-service and in-service training and assessment for supervisors each year. More measures are created to make the training fruitful, e.g., the application of moral education, the development of supervisors’ responsibilities, the acquaintance with educational policies and administrative systems related to Ph.D. students, the observation of academic ethics and norms, the tips of supervisors’ physical and mental self-adjustment, etc. All this is conducted in various forms, such as, experts’ demonstrations, peer communication and seminars. It will effectively provide the basic, but powerful supervisory skills (both professional and personal) and responsibilities that all supervisors must have in order to understand and connect with the students and drive results, integrating a theory of “becoming a supervisor” into supervisor professional development.

(5) Providing supervisors with a continuum of school mental health services

It is vital to offer students access to a continuum of free, confidential school mental health services to effectively address issues directed to supervisors’ work life and safeguard supervisor well-being [61]. It should be pointed out to supervisors that seeking help is not a sign of weakness. After all, teaching is surprisingly one of the most stressful jobs in the United States [62], and perhaps this is even more the case in China. So, supervisors should be encouraged to participate in various amateur activities and communicate with their friends, family members and colleagues in their spare time, which is beneficial to keep a balanced interpersonal relationship and obtain more support from their universities, the community and society. Research also reveals that in addition to the positive benefits of improved teacher job satisfaction, health and well-being, there are documented cost savings and impacts on student outcomes related to having healthy teachers and school staff [63].

## 5. Limitations

There are three main limitations of this research. Firstly, selection of participants was limited to certain regions, and the sample size was rather small, not indicative of the large population of supervisors across China. Secondly, further analysis should be conducted to cover different levels of stress suffered by participants due to their personal experiences. Thirdly, use of WeChat for data gathering methods tremendously limited the amount of non-verbal data which could have been gathered otherwise, thus limited the probes and follow-up questions concerning the positive or negative influence on the well-being of supervisors following the reforms in higher education.

## 6. Conclusions

This study brings to light just how stressful the job of doctoral supervision can be, cohering with yet contributing to the emerging and growing body of literature on the psychological health crisis prevalent in higher education. More importantly, this study applied grounded theory to reveal varied factors of academic and interpersonal nature for occupational stress that the participants experienced as doctoral supervisors, and to present a substantive theory that explains the relationship between a variety of related conditions.

Hopefully, this research can provide some insight for certain reforms in respect to doctoral education, arouse concerns from universities and institutions about the psychological health of this special group of people, facilitate the promulgation of related guidelines to alleviate the distressing symptoms among supervisors and promote sustainable development in higher education.

This qualitative study conducted semi-structured interviews to explore the perspectives of the supervisors through inviting them to share their own stories, as it is their perceptions and experiences which are of interest here. Thus, more strategies can be put forward to cope with their stress based on their respective experiences.

## Figures and Tables

**Table 1:** Interview guide.

Order	Questions
1	What comes to your mind when “stress” is mentioned?
2	Do you think stress is prevalent among doctoral supervisors? If so, give examples;
3	During your life time as a doctoral supervisor, have you ever suffered from any stress? If so, what are the main stressors?
4	Have you received any training with respect to addressing mental health issues before you are elected as a doctoral supervisor? Is the training necessary? What, if any, are the positive aspects of the training?
5	In respect to the stressors, what suggestions would you propose to alleviate the distressing symptoms among supervisors?
6	Is there anything else you would like to add?

**Table 2:** Coding of identified occupational stress suffered by Chinese doctoral supervisors.

Paradigm	Categories	Subcategories
Causal conditions	Demanding academic requirements	Publication of 3 or more articles in key journals within 3 or 4 years
		Hosting a research program at ministerial level or above
	Heavy teaching workload	Annual workloads ranging from 300 to 340 credit hours in two semesters
	Various student-related issues	Student graduation with article publication and thesis completion
		Negative relationship between supervisors and Ph.D. students
		Constraint of new Ph.D. student recruitment
Central phenomenon	Experience of stress	Negative emotions (dissatisfaction, frustration, exhaustion, loss of face etc.)
Contextual conditions	Policies and regulations in higher education	Unreasonable performance appraisal
		Complex student-related issues
Intervening conditions	Personal performance appraisal	Difficulties in article publication
		Requirements for awards and programs
	Doctoral student-related issues	Tensed relationship between supervisors and Ph.D. students
		Various *guanxi* involved in the recruitment of Ph.D. students (*Guanxi* (关系), in Chinese culture, means the system of social networks and influential relationships which facilitate business and other dealings. *Guanxi* is often translated as “connections”, “relationships” or “networks”.).
Action/Interaction strategies	Working with long hours	Academic research (article publication, hosting/winning research programs and awards)
		Teaching workload
	Self-compromise	Dealing with student-related issues
Consequences	Experiencing various stressors	A single stressor
		Combination of a set of stressors

## Data Availability

The data presented in this study are not publicly available due to privacy concerns.
